# Plastid Genome Sequence of a Wild Woody Oil Species, *Prinsepia* *utilis*, Provides Insights into Evolutionary and Mutational Patterns of Rosaceae Chloroplast Genomes

**DOI:** 10.1371/journal.pone.0073946

**Published:** 2013-09-02

**Authors:** Shuo Wang, Chao Shi, Li-Zhi Gao

**Affiliations:** 1 Faculty of Life Science and Technology, Kunming University of Science and Technology, Kunming, China; 2 Plant Germplasm and Genomics Center, Germplasm Bank of Wild Species in Southwest China, Kunming Institute of Botany, the Chinese Academy of Sciences, Kunming, China; 3 University of the Chinese Academy of Sciences, Beijing, China; University of Arizona, United States of America

## Abstract

**Background:**

*Prinsepia* *utilis* Royle is a wild woody oil species of Rosaceae that yields edible oil which has been proved to possess particular benefits for human health and medical therapy. However, the lack of bred varieties has largely impeded exploiting immense potentials for high quality of its seed oil. It is urgently needed to enlarge the knowledge of genetic basis of the species and develop genetic markers to enhance modern breeding programs.

**Results:**

Here we reported the complete chloroplast (cp) genome of 156,328 bp. Comparative cp sequence analyses of *P* *. utilis* along with other four Rosaceae species resulted in similar genome structures, gene orders, and gene contents. Contraction/expansion of inverted repeat regions (IRs) explained part of the length variation in the Rosaceae cp genomes. Genome sequence alignments revealed that nucleotide diversity was associated with AT content, and large single copy regions (LSC) and small single copy regions (SSC) harbored higher sequence variations in both coding and non-coding regions than IRs. Simple sequence repeats (SSRs) were detected in the *P* *. utilis* and compared with those of the other four Rosaceae cp genomes. Almost all the SSR loci were composed of A or T, therefore it might contribute to the A-T richness of cp genomes and be associated with AT biased sequence variation. Among all the protein-coding genes, *ycf1* showed the highest sequence divergence, indicating that it could accomplish the discrimination of species within Rosaceae as well as within angiosperms better than other genes.

**Conclusions:**

With the addition of this new sequenced cp genome, high nucleotide substitution rate and abundant deletions/insertions were observed, suggesting a greater genomic dynamics than previously explored in Rosaceae. The availability of the complete cp genome of *P* *. utilis* will provide chloroplast markers and genetic information to better enhance the conservation and utilization of this woody oil plant.

## Introduction

Rosaceae, containing about 3,000 species in over 100 genera, is the third economically important plant family in temperate regions [1]. A vast majority of common fruits, including *Malus* (apples), *Pyrus* (pears), *Prunus* (plums, peaches, cherries, almonds, and apricots), *Rubus* (raspberries), and *Fragaria* (strawberries), as well as plenty sources of ornamentals, e.g., *Rosa* (roses), *Potentilla* (cinquefoils), and *Sorbus* (mountain ashes) are produced by members of this family. From the taxonomical perspective, Rosaceae has traditionally been divided into four subfamilies: Rosoideae (fruit, achene), Prunoideae (fruit, drupe), Spiraeoideae (fruit, follicle or capsule) and Maloideae (fruit, pome) on the basis of fruit type [1,2]. While it was classified into three to twelve subfamilies historically based on molecular phylogenetic studies [1,3]. The economic importance of rosaceous crops has drawn several genome sequencing projects to these species. To date, five nucleic genomes, *Fragaria vesca* (strawberry) [4], *Malus*×*domestica* (apple) [5], *Pyrus* *bretschneideri* (pear) [6], *Prunus persica* (peach), and *P* *. mume* [7], were completely sequenced. Compared with the availability of so many nucleic genome sequences, our understanding of Rosaceae’s cp genomes is left behind, as only five cp genomes in this family (strawberry [4], apple [5], pear [8], peach [9], and *P* *. rupicala* ) have been sequenced so far. Thus Rosaceae stands out as a worldwide distributed and economically important but cp genome-wide limited and taxonomically ambiguous family of angiosperms.

*Prinsepia* *utilis* , one of the major woody oil plants in Rosaceae, is mainly distributed in mountainous regions with high elevations of 1000-3200 m in southwestern China, Pakistan, India, Nepal and Bhutan[10,11]. . In addition to *P* *. utilis* , there are the other three related species, *P* *. sinensis* (Oliv.) Oliv. ex Bean, *P* *. uniflora* Batal, and *P* *. scandens* Hayata, in the genus *Prinsepia* . Seed kernels of *P* *. utilis* averagely yield 37.2% of semi-drying and pale yellow fatty oil which has widely been consumed for cooking [10]. Up to date, the chemical components of its seed oil (e.g., oleic acid 32.6%; linoleic acid 43.6%; palmitic acid 15.2%) have been proved to possess particular benefits or potentials for human health and medical therapy [12,13], which can be undoubtedly comparable with Mediterranean olive oil. It is well known that the majority of woody oil plants grow in tropical and subtropical regions. *P* *. utilis* may represent as the best candidate oil plant which is suitable for extensive cultivation with cold weather in higher altitudes of temperate regions. The lack of bred varieties in *P* *. utilis* , however, has largely impeded exploiting immense potential for high quality of seed oil. Nowadays, the collection and transplanting of wild plants have seriously destroyed natural populations, making continuous loss of numerous useful germ plasm resources. It is urgently needed to enlarge the knowledge of genetic basis of the species and develop genetic markers that will be fairly necessary to enhance modern breeding programs for the purpose of the selection and breeding of new varieties.

The affinity of *P* *. utilis* has been argued by many botanists since the species was reported in 1839 [14]. Based on morphological characteristics, Royle (1839) and Lindley (1853) assigned this newly described species to the tribe Chrysobalanoideae [15,16], Bentham and Hooker (1862-1867) placed it in the tribe Prunoideae [17], while Sterling (1963) assigned it to a new subfamilial group in Prinsepioideae, Rosaceae [14]. Its subfamilial characters are carpel solitary, style lateral or sub-basal; 2 ovules erect and campylotropous, the nucelli horizontal, micropyles directed toward the ventral obturator; fruit with leathery pit [14]. In recent years, it has been placed into Osmaronieae (supertribe kerriodae and subfamily Spiraeoideae) with molecular phylogenetic support [1].

Chloroplasts were derived from endosymbiosis between independent living cyanobacteria and a non-photosynthetic host [18]. This intracellular organelle encodes a number of chloroplast-specific components and has high levels of conservation [19]. Chloroplast DNA (cpDNA) of green plants provides sufficient information for genome-wide evolutionary studies. It has shown great potentials in addressing phylogenetic questions at both high and low taxonomic levels, and sometimes it is necessary to use complete cp genome sequences for resolving complex evolutionary relationships [20–22]. However, acquiring large coverage of cp genomes has typically been limited by conventional DNA sequencing. As next-generation sequencing techniques have revolutionized DNA sequencing via high-throughput capabilities and relatively low costs [23], it is now more convenient to obtain cp genome sequences and extend gene-based phylogenetics to phylogenomics.

In this study, we sequenced the complete cp genome sequence of a wild woody oil plant, *P* *. utilis* , which boasted great economic and taxonomic values in Rosaceae. Comparative analyses of the five Rosaceae plastomes, including the *P* *. utilis* plastome sequenced in this study, were further performed to provide insights into overall and especially evolutionary dynamics of Rosaceae cp genomes by detecting genome-wide sequence variations.

## Results and Discussion

### Genome organization of P. utilis

**Figure 1:**
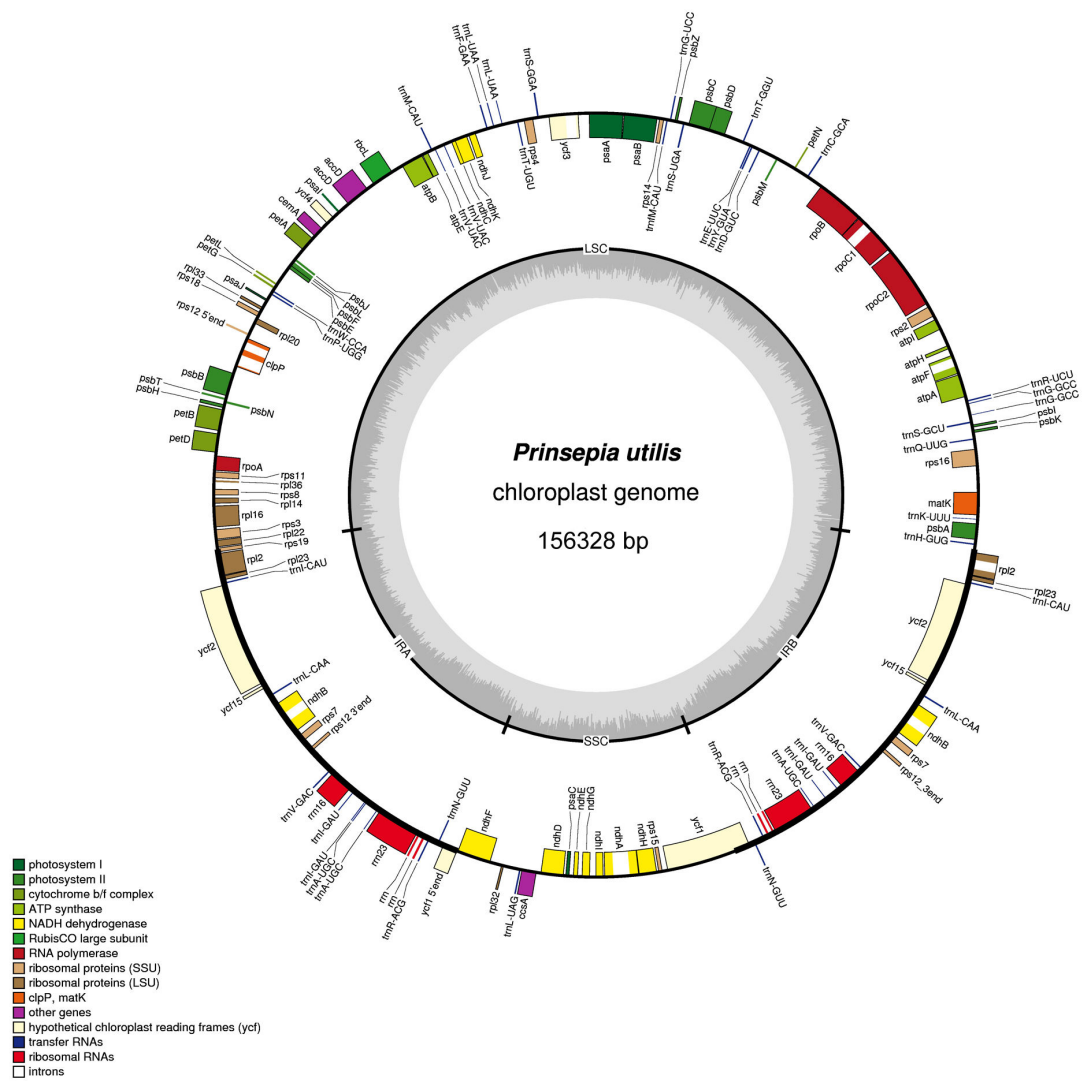
Gene map of the *P* *. utilis* chloroplast genome. Genes lying outside of the outer circle are transcribed in the clockwise direction whereas genes inside are transcribed in the counterclockwise direction. Genes belonging to different functional groups are color coded. Area dashed darker gray in the inner circle indicates GC content while the lighter gray corresponds to AT content of the genome.

The cp genome of *P* *. utilis* exhibited a circular DNA molecule of 156,328 bp with a typical quadripartite structure resembling to the majority of land plant cp genomes. It consisted of two inverted repeat regions (IRa and IRb) of 26,302 bp separated by large (LSC) and small (SSC) single-copy regions of 85,239 and 18,485 bp, respectively (Figure 1). The cp genome encoded 112 unique genes, 19 of which were duplicated in the IR regions, giving a total of 131 genes (Figure 1, Table 1). Among these unique genes, 18 included one or two introns. All of these coding regions accounted for 51.3% of the whole genome. The gene contents and gene order of *P* *. utilis* were identical to those of the four previously known Rosaceae plastomes (Table 2).

**Table 1:** List of genes encoded by *P* *. utilis* chloroplast genome.

Category for genes	Group of gene	Name of gene
Self replication	Large subunit of ribosome	rpl2*, *rpl14*, rpl16*, *rpl20*, *rpl22*, *rpl23*, *rpl32*, *rpl33*, *rpl36*
	Small subunit of ribosome	*rps2*, *rps3*, *rps4*, *rps7*, *rps8*, *rps11*, *rps12*, *rps14*, *rps15*, rps16*, *rps18*, *rps19*
	DNA dependent RNA polymerase	*rpoA*, *rpoB*, rpoC1*, *rpoC2*
	rRNA genes	*rrn*4.5, *rrn*5, *rrn*16, *rrn*23
	tRNA genes	trnA-UGC*, *trnC-GCA*, *trnD-GUC, trnE-UUC*, *trnF-GAA*, *trnG-UCC*, trnG-GCC*, *trnH-GUG*, *trnI-CAU*, trnI-GAU*, trnK-UUU*, *trnL-UAG*, *trnL-CAA*, trnL-UAA*, *trnM-CAU*, *trnfM-CAU*, *trnN-GUU*, *trnP-UGG*, *trnQ-UUG*, *trnR-ACG*, *trnR-UCU*, *trnS-GGA*, *trnS-GCU*, *trnS-UGA*, *trnT-GGU*, *trnT-UGU*, trnV-UAC*, *trnV-GAC*, *trnW-CCA*, *trnY-GUA*
Photosynthesis	Subunits of photosystemⅠ	*psaA*, *psaB*, *psaC*, *psaI*, *psaJ*, ycf3**, *ycf4*
	Subunits of photosystemⅡ	*psbA*, *psbB*, *psbC*, *psbD*, *psbE*, *psbF*, *psbH*, *psbI*, *psbJ*, *psbK*, *psbL*, *psbM*, *psbN*, *psbT*, *psbZ*
	Subunits of NADH-dehydrogenase	ndhA*, ndhB*, *ndhC*, *ndhD*, *ndhE*, *ndhF*, *ndhG*, *ndhH*, *ndhI*, *ndhJ*, *ndhK*
	Subunits of cytochrome b/f complex	*petA*, petB*, petD*, *petG*, *petL*, *petN*
	Subunits of ATP synthase	*atpA*, *atpB*, *atpE*, atpF*, *atpH*, *atpI*,
	Large subunit of rubisco	*rbcL*
Other genes	Translational initiation factor	*infA*
	Maturase	*matK*
	Protease	clpP**
	Envelop membrane protein	*cemA*
	Subunit of Acetyl-CoA-carboxylase	*accD*
	c-type cytochrom synthesis gene	*ccsA*
Genes of unknown function	Conserved Open Reading Frames (ORF, *ycf*)	*ycf1*, *ycf2*

One and two asterisks indicate one- and two-intron containing genes respectively

**Table 2:** Summary of the Rosaceae chloroplast genome features.

	** *P* *. utilis* **	***P. persica***	** *P* *. pyfifolia* **	** *P* *. rupicola* **	***F. vesca***
**Genome size (bp)**	156,328	157,790	159,922	156,612	155,691
**LSC length (bp)**	85,239	85,968	87,901	84,970	85,606
**SSC length (bp)**	18,485	19,060	19,237	18,942	18,175
**IR length (bp)**	26,302	26,381	26,392	26,350	25,555
**Number of genes**	131	131	131	131	131
**Percent of coding regions**	51.3%	50.8%	50.1%	51.2%	51.5%

From the aspect of genome size, *P* *. utilis* , next to *F. vesca* (155,691 bp), is the second smallest one among the five sequenced Rosaceae cp genomes. It is about 3.6 kb, 1.5 kb, and 0.3 kb smaller than *P* *. pyfifolia* , *P. persica*, and *P* *. rupicola* , respectively. The genome size variation can be explained mainly by differences in the length of SSC and IR regions (Table 2).

### Contraction/expansion of IRs among Rosaceae cp genomes

**Figure 2:**
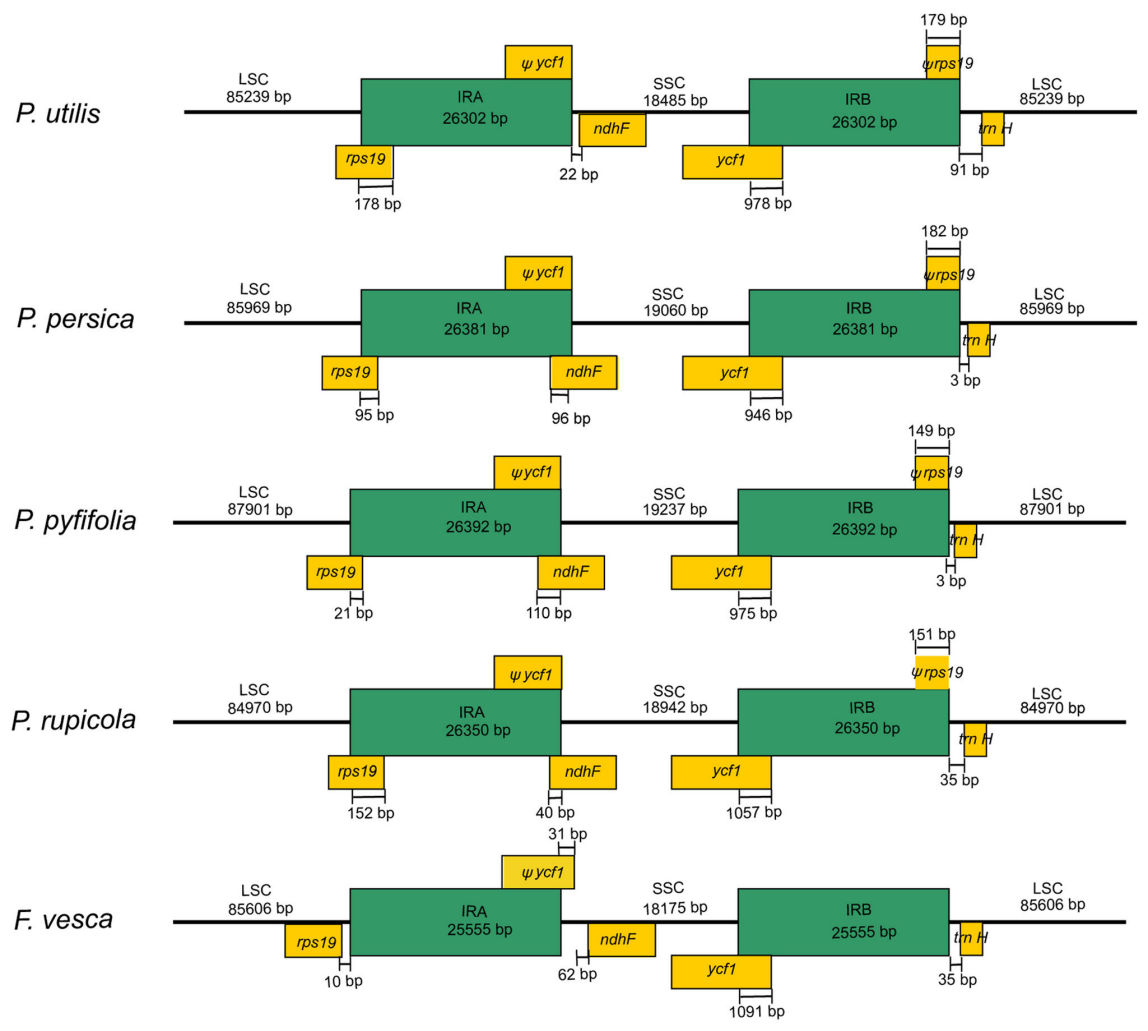
Detailed view of the IR junctions among the five Rosaceae chloroplast genomes. Annotated genes or portions of genes are indicated by yellow boxes above or below the genome.

Generally, the lengths of IR (IRa and IRb) regions differ among various plant species. Contraction or expansion of the IR regions often results in length variation of cp genomes [24]. In this study, the detailed IR–SSC and IR-LSC borders, together with the adjacent genes, were compared across the five Rosaceae cp genomes (Figure 2). Of all the species except *F. vesca*, the junction of LSC and IRa was located in the coding region of *rps19*. The end of IRa of *P* *. utilis* expanded 178 bp into *rps19*, creating a truncated *rps19* pseudogene of the same length at the IRb-LSC border. A similar feature was also observed in the other three cp genomes that the end of IRa expanded 152 bp, 95 bp, and 21 bp in *P* *. rupicola* , *P. persica* and *P* *. pyfifolia* , respectively. In *F. vesca*, LSC region possessed an intact *rps19* gene and expanded to incorporate 10 bp non-coding regions apart from the IRa-LSC border. On the boundary of IRa and SSC, IRa of all the species expanded to *ycf1* and resulted in a truncated *ycf1* gene with the same length as its homologous part in IRb. In *P* *. rupicola* , *P. persica*, and *P* *. pyfifolia* , a part of the *ndhF* gene was located in IRa; in *P* *. utilis* and *F. vesca*, the entire *ndhF* gene was located in SSC region with 22 bp and 93 bp apart from the IRa–SSC border, respectively. A similar pattern of expansion was found in IRb. In all five species, the initiation of IRb partially contained the *ycf1* gene, which was terminal with the *rps19* pseudogene. In *F. vesca*, the nonfunctional *rps19* gene seemed to be lost. Overall, a similar pattern of contraction/expansion was observed when these cp genomes approximately had a comparable genome size, for example, between *P* *. utilis* and *F. vesca*, and among the other three cp genomes.

### Genome-wide sequence variations among Rosaceae cp genomes

**Figure 3:**
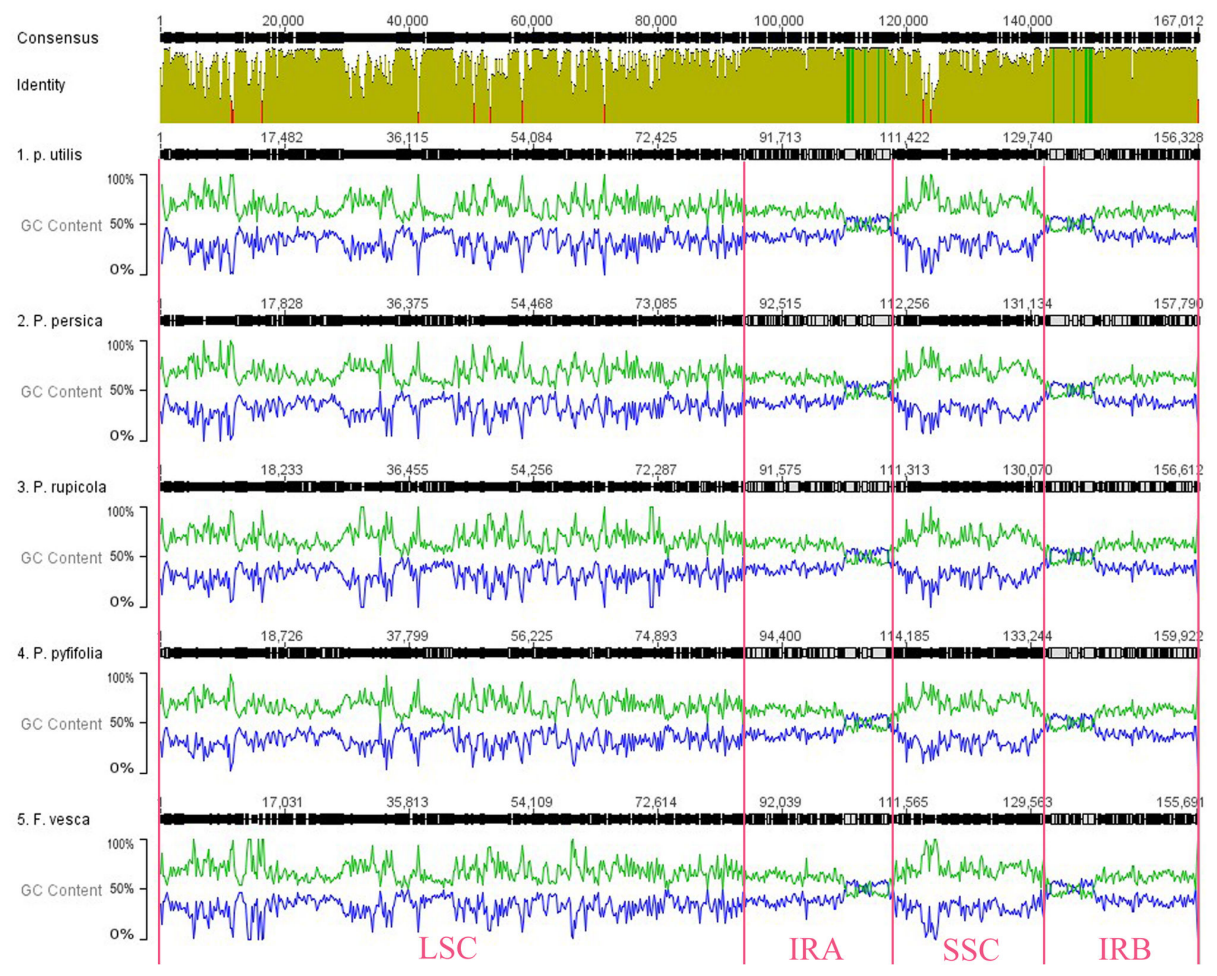
Whole chloroplast genome alignment of the five Rosaceae species with **GC**/**AT** content plotted along the sequences. The alignment was wrapped corresponding to sequence similarity and GC/AT content was calculated in every 100 bp sequence window. The green lines indicate AT content, while the blue lines refer to GC content.

We first aligned them using MAFFT [25] to identify genome-wide sequence variation among these five Rosaceae cp genomes (as shown in Figure 3). As previously studied [26–28], most of the sequence variations were located in LSC and SSC regions, while IR regions exhibited comparatively fewer sequence variations. Interestingly, a positive association between A/T content and sequence divergence was observed when base content was introduced into sequence alignment. Since IR region of cp genomes harbors lower A/T content, this might have led to higher sequence conservation of this region. Furthermore, the low A/T content of rRNA genes (above 50%) was also consistent with its high sequence conservation (data not shown). It has been well known that cp genomes of plants are quite A/T rich (above 60%) [28], and thus base content may be a major reason for determining cp genome variations.

To look sequence divergence into details, we further plotted sequence divergence along the whole genomes with genes annotated (Figure S1). Consistent with other angiosperms, sequence divergence in intergenic regions was higher than that in genic regions of these five genomes. Intergenic regions with higher levels of divergence were *matK–rps16*, *rps16–psbK*, *psbI–atpA*, *atpH–atpI*, *petN–psbM*, *ndhC–atpE*, and *rpl32–ccsA*. Genic regions were *ycf1*, *rpl22*, and *matK*. The large sequence divergence in both coding and non-coding regions was mainly due to indels, as the sites where large indels (≥100 bp) occurred were mostly located in LSC or SSC regions. In addition, the IR–SSC and IR-LSC borders also harbored more sequence variations, which coincided with quite often contraction or expansion of the IR regions.

### Simple sequence repeats (SSRs) of Rosaceae cp genomes

Cp SSRs have been proven to be highly valuable in plant population genetic [29,30] and phylogenetic studies [31,32]. In this study, we calculated the number of SSRs of *P* *. utilis* together with that of the previously sequenced four Rosaceae cp genomes by using thresholds of mononucleotide repeats ≥ 8 nt, dinucleotide repeats ≥ 5 nt, and trinucleotide repeats ≥ 5 nt. We also calculated the number of longer repeats with thresholds of mononucleotide repeats ≥ 12 nt, dinucleotide repeats ≥ 10 nt, and trinucleotide repeats ≥ 10 nt [33]. There is no particular criterion about which nucleotide length or repeat unit may have a significant variation [34]. However, it has been suggested that SSRs longer than 8 nt tend to cause slip-strand mispairing during DNA replication [35], which is generally acknowledged as the major mechanism by which length variants are generated [36,37]. Based on the first criteria, we found 182 (168+14+0), 176 (159+17+0), 199 (180+19+0), 174 (166+8+0), 161 (147+13+1) 8, 5, 5 SSRs in *P* *. utilis* , *P. persica*, *P* *. pyfifolia* , *P* *. rupicola* , and *F. vesca*, respectively. While according to the second criteria, only 21, 21, 35, 19 and 9 mononucleotide repeats were observed in these five species, respectively (Table 3).

**Table 3:** Number of SSRs present in Rosaceae chloroplast genomes.

	**8, 5, 5, SSRs (number)**						**12, 10, 10 SSRs (number)**					
**Taxon**	**mono**	**di**	**tri**	**total**	**A or T mono. repeat**	**coding region**	**mono**	**di**	**tri**	**total**	**A or T mono. repeat**	**coding region**
** *P* *. utilis* **	168	14	0	182	177	55	21	0	0	21	21	4
	(92%)	(8%)			(97%)	(31%)	(100%)				(100%)	(19%)
***P. persica***	159	17	0	176	171	47	21	0	0	21	21	2
	(90%)	(10%)			(97%)	(36%)	(100%)				(100%)	(10%)
** *P* *. pyfifolia* **	180	19	0	199	195	42	35	0	0	35	35	1
	(90%)	(10%)			(98%)	(21%)	(100%)				(100%)	(3%)
** *P* *. rupicola* **	166	8	0	174	160	44	19	0	0	19	19	1
	(95%)	(5%)			(96%)	(26%)	(100%)				(100%)	(5%)
***F. vesca***	147	13	1	161	152	35	7	2	0	9	9	0
	(91%)	(8%)	(1%)		(94%)	(21%)	(78%)	(22%)			(100%)	

Among these cpSSR nucleotide units, the longest repeat stretch was polyA and (or) polyT with 16–17 bp, and most of the repeat units (> 95%) were also composed of polyA or polyT in these cp genomes; when the threshold was increased, all the repeat units were composed of polyA or polyT (Table 3). This was consistent with the observation in previous studies that cpSSRs were dominated by A or T mononucleotide repeats [38]. Furthermore, cpSSRs were unevenly distributed along the whole genome with about 70% in noncoding regions (Table 3). The composition and distribution of cpSSRs were quite similar within these Rosaceae cp genomes. Among these identified cpSSRs, about 80% were shared among these five Rosaceae cp genomes (similar repeat units located in similar genomic regions) (data not shown), suggesting the potential of these cpSSRs to be applied in further studies of more Rosaceae species.

Here, we found that most of the cpSSRs were A or T mononucleotide repeats, and they were mainly located in intergenic regions which harbored the vast majority of genomic variation. Previous studies also suggested that most repeats and indels were located in intergenic regions which were A + T-rich in *Trifolium* cp genome [39]. Therefore, the observation that the genome divergence was associated with AT content in this study may indicate a common mutational pattern in the cp genomes of angiosperms.

### Phylogenetic analysis based on the complete cp genome sequences

To explore phylogenetic relationship of *P* *. utilis* with other major angiosperms clades, we employed protein-coding sequences to resolve relationships among these divergent angiosperm clades. Since our interest focused on phylogenetic relationship between Rosaceae and its close sister clades, with an especial emphasis on its relationship with other species of rosids, we included most of the angiosperms clades previously studied instead of all the sequenced cp genomes [20,40]. A dataset including 78 protein-coding genes comprised of 65,713 aligned nucleotide characters was finally used for phylogenetic analyses of 78 taxa.

**Figure 4:**
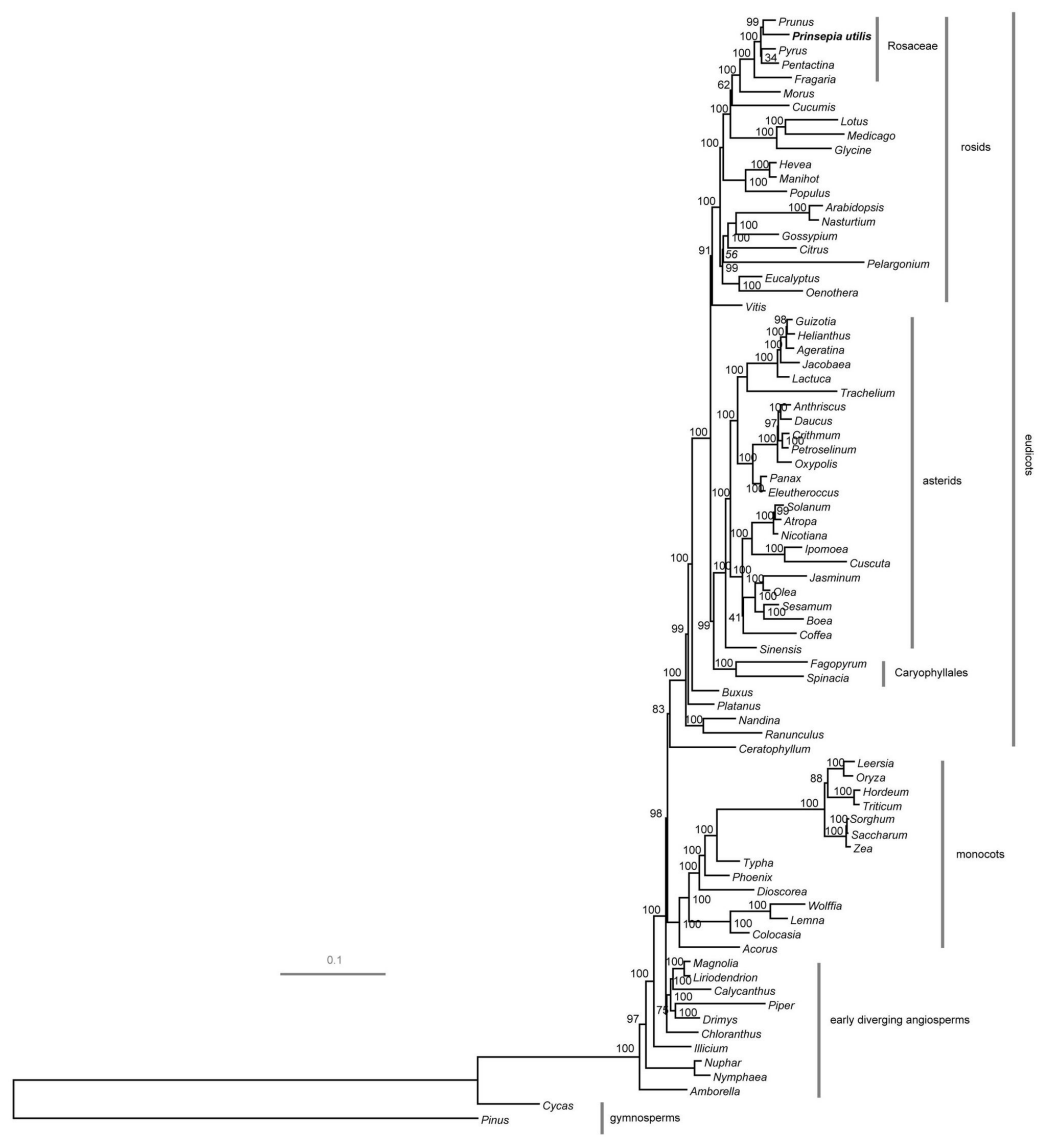
ML phylogram of the angiosperms using whole chloroplast genome sequences. The tree has two outgroup taxa, *Pinus* *thunbergii* and *Cycas* *taitungensis* . Numbers above each node indicate the ML bootstrap support values.

Maximum likelihood (ML) analysis resulted in a single tree with -lnL = 833985.25 (Figure 4). Bootstrap analyses indicated that most of the nodes (67/74) were supported by values of 95% or greater and 57 of these had a bootstrap value of 100%. Maximum parsimony (MP) analysis also generated a single, fully resolved tree with a length of 74,348, a consistency index of 0.354 (excluding uninformative characters) and a retention index of 0.614 (Figure S2). Bootstrap analyses indicated that 58 of the 78 nodes were supported by values of 95% or greater, and 56 of these had a bootstrap value of 100%. Both the ML and MP trees produced a similar topology (Figure 4 and Figure S2) and one of major differences concerned the position of *Ceratophyllum* as described in earlier papers [20,40]. The major clades including basal angiosperms, monocots, eudicots were strongly supported in this study.

In Rosaceae, both ML and MP analyses strongly supported that *F. vesca* was sister to the rest of the four species. Besides, ML tree divided the rest four species into two sister clades with 100% bootstrap support. The first clade included *P* *. utilis* and *P. persica*, and the second clade was consisted of the other two species. MP tree made a little difference that *P* *. utilis* and *P. persica* were grouped together but *P* *. pyfifolia* and *P* *. rupicola* did not form one sister clade. By comparing with previous studies, ML tree might represent a more correct topology in constructing Rosaceae phylogeny by using these genes [1,2]. Thus, only the ML tree was included in our latter analyses.

**Figure 5:**
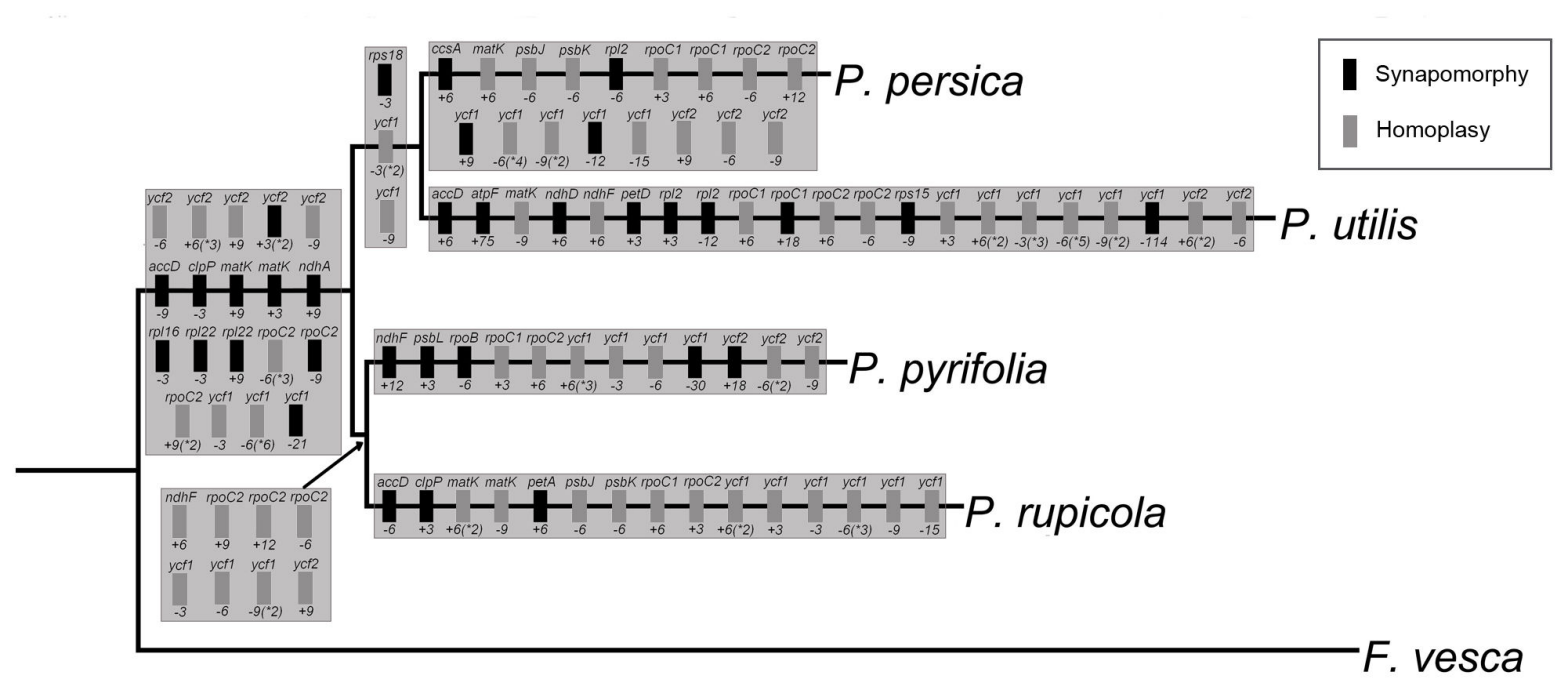
Phylogenetic distribution of exon coding indels in the five Rosaceae chloroplast genomes. The phylogenetic tree was a subtree of the one in Figure 4. Gene names are given above boxes, sizes of indels (bp) and polarity (‘+’ = insertion, ‘-’ = deletion) are given below boxes, and numbers following * in parentheses indicate indels which appeared no less than twice in one gene. Polarity of mutations was determined by comparison to the outgroup, *F*. *vesca*.

To identify indel variations along the Rosaceae phylogeny, a total of 95 indels in the gene regions of which the largest gap size was over 110 bp were found and mapped to the cp genome-based phylogenetic tree using *F. vesca* as an outgroup (Figure 5). These 95 indels were distributed in 23 different genes, three (*rpoC2*, *ycf2*, and *ycf1*) of which contained as many as 12, 14, and 27 indels, respectively. Among these 7 branches, the branch leading to *P* *. utilis* contained most of the indels (21 of 95). The second most were contained by the common branch leading to two sister clades (19 of 95). The branch leading to the clade of *P* *. utilis* and *P. persica* only harbored three indels. Up to now, there is no clear consensus about whether indels are useful or not in phylogenetic analyses [41,42], and the doubtfulness is essentially based upon studies in which only one or several DNA fragments were utilized. In this study, 33 of all the indels observed were unambiguously mapped to monophyletic groups and they might have intensive benefit for nucleotide-based phylogenetic analyses. The remained indels might be homoplasies and possibly had negative effects on the reconstruction of phylogenetic tree. Previous studies also suggest that indels should be cautiously used in phylogenetic studies especially when few DNA fragments were analyzed.

### Identification of chloroplast molecular markers

Since cp genomes of higher plants are quite conserved, the identification of molecular markers with higher sequence variation could be fairly valuable for population genetic and phylogenetic studies. Our genome-wide comparative analyses found that some genes boasted superior sequence divergence which could be definitely suitable for phylogeny studies (Figure S1). The values of overall mean distance (standing for genetic divergence) of 76 protein-coding genes in the five Rosaceae cp genomes, were further calculated (Figure S3). Among them, *ycf1*, one of the largest genes in the cp genomes (about 5.5 kb), harbored the highest sequence divergence. To explore its phylogenetic utility, Maximum likelihood (ML) analysis for *ycf1* was conducted in 65 species resulting in a single tree with -lnL = 179121.15 and high bootstrap values (Figure S4). 37 of the 62 nodes possessed bootstrap values of 95% or greater and 31 of which had 100%. Despite the limited number of characters, overall topology of the phylogeny was quite similar to the one constructed with 78 protein-coding genes (Figure 4), indicating that *ycf1* showed the potential for phylogenetic analysis in angiosperms. By comparing with another 9 potential molecular markers of which sequence divergence was no lower than 0.65 (*rps18*, *rpl32*, *ccsA*, *ndhF*, *matK*, *rpl22*; Figure S3) or indels were no less than 6 (*matK*, *rpoC1*, *rpoC2*, *ycf2*; Figure 5), *ycf1* still performed the best to differentiate these five Rosaceae species (Figure S5). While other markers presented diverse topologies in reconstructing phylogeny of Rosaceae and with lower node supports, *ycf1* showed the same topology as the subtree constructed by 78 protein-coding genes with comparatively higher node supports (Figure S5). Thus, *ycf1* may serve as the best gene marker in distinguishing phylogenetic relationships within both Rosaceae and angiosperms.

Finding variable and easily aligned DNA sequences has been a challenging task in molecular genetics. Until now, most of DNA sequences used in plant phylogenetic analysis have come from the plastid genes, e.g. *matK*, *rbcL*, and *trnL-F* which have been extensively used. Although utility of these DNA regions has often obtained well-resolved phylogenies, the need for more data to address questions at multiple phylogenetic levels still persists. Previous studies indicated that *ycf1* may be the most variable coding region in the cp genome, which is consistent with our analysis in the Rosaceae [43–45]. The usage of *ycf1* has been confined to only orchids [43], *Pinus* [44], and magnoliid [45] where it has been demonstrated to be phylogenetically useful in analyses at the species, genus, and subfamily levels. Our study cans broad its uses as a new marker at least in Rosaceae.

## Conclusion

In this study, we reported the complete chloroplast genome sequence of *P* *. utilis* , a wide woody oil plant in Rosaceae. The plastome was characterized by comparing with other sequenced plastomes in Rosaceae and the evolutionary position of *P* *. utilis* in Rosaceae was determined by phylogenetic analysis of concatenated alignments of gene sequences. While comparisons of plastomes in Rosaceae have identified many regions with higher sequence divergence, *ycf1* was proved to boast the potential to solve phylogenetic issues ranging from lower taxonomic ranks to the relationships among genera and species. The availability of the complete cp genome of *P* *. utilis* will provide new resources for the utilization of cp genome sequence to further evolutionary studies and genetic breeding programs in Rosaceae.

## Materials and Methods

### Ethics statement

The species is naturally grown in Kunming Botanical Garden of Kunming Institute of Botany, the Chinese Academy of Sciences. The voucher specimens were deposited at the Herbarium of Kunming Institute of Botany (KUN). Permit for collection of the plant leaves was obtained from Wei-bang Sun, Kunming Botanical Garden, the Chinese Academy of Sciences.

### Chloroplast DNA Extraction, Genome Sequencing and Assembly

About 20g fresh mature leaves were sampled from a single *P* *. utihis* plant. cpDNA was extracted by use of a modified high salt method reported by our group [46].

After the cpDNA isolation, approximately 5-10µg of DNA was sheared, followed by adapter ligation and library amplification, and then subjected to Illumina Sample Preparation Instructions. The fragmented cpDNAs were sequenced at both single-read using the Illumina Genome Analyzer IIx platform at the in-house facility at The Germplasm Bank of Wild Species in Southwestern China. Files containing the raw read sequences are available from the National Center for Biotechnology Information (NCBI) Short Read Archive with the accession number: SRX277333. The obtained paired-end reads (2×100 bp read lengths) were assembled using SOAP de novo [47] to the cp genome sequence of *P. persica*, which was downloaded from GenBank (NC_014697) and served as a reference genome. Regions with ambiguous alignment (conflicted reads mapped to the same genomic region) were trimmed off manually and considered as gaps. Polymerase chain reaction (PCR) amplified fragments yielded by primers (Table S1) derived from the terminal ends of contigs, and the fragments were then sequenced to flank the gap regions. The PCR amplification reactions were template denaturation at 80°C for 5 min followed by 30 cycles of denaturation at 95°C for 30 sec, primer annealing at 55°C for 30 sec, and primer extension at 65°C for 1 min; followed by a final extension step of 5 min at 65°C [48]. PCR products were separated by electrophoresis in 1.5% agarose gel and sequenced on an Applied Biosystems (ABI) 3730 automated sequencer. Finally, the average coverage depth reached approximately 855×, which is greatly higher than the proposed minimum for cpDNAs (30×) [49].

### Genome Annotation

Annotation of the assembled genome was performed with Dual Organellar GenoMe Annotator (DOGMA) using default parameters to predict protein-coding genes, transfer RNA (tRNA) genes, and ribosome RNA (rRNA) genes [50]. BLASTX and BLASTN searches against a custom database of previously published cp genomes identified locations of these putative genes. For genes with low sequence identity, manual annotation was performed to determinate the position of start and stop codons depending on the translated amino acid sequence using the chloroplast/bacterial genetic code. Gene map of this plastome was drawn by OGDraw v 1.2 [51]. The plastid genome sequence has been deposited in GenBank (accession number KC571835).

### Whole-genome Sequence Alignment and Detection of SSRs

mVISTA program (http://genome.lbl.gov/vista/index.shtml) was utilized for pair-wise whole-genome alignment of the five Rosaceae cp genomes with *F. vesca* serving as an outgroup. This program takes both fasta format sequences and gene annotation text as input files, but here, only *F. vesca* gene annotation was used. To further estimate sequence divergences, the common protein-coding genes were extracted from these five species and aligned in MEGA5 [52]. Gene divergences represented by overall mean distance were calculated with Jukes-cantor model with all gaps and missing data treated as complete deletion. SSRs were detected using Msatfinder on-line v.2.0 (http://www.genomics.ceh.ac.uk/cgi-bin/msatfinder/msatfinder.cgi). We calculated the number of SSRs by using two thresholds of 1) mononucleotide repeats ≥ 8 nt, dinucleotide repeats ≥ 5 nt, and trinucleotide repeats ≥ 5 nt; 2) mononucleotide repeats ≥ 12 nt, dinucleotide repeats ≥ 10 nt, and trinucleotide repeats ≥ 10 nt.

### Phylogenetic Analyses

For the purpose of phylogenetic analyses, two data sets were utilized. The first set included 78 common protein-coding genes which were extracted from 76 angiosperm cp genomes and 2 gymnosperm outgroups, representing different plant lineages of which cp genomes were sequenced (see Table S2 for a complete list of the genomes examined). For all the species, every gene was translated into amino acid sequence separately, and then aligned in MSWAT (http://mswat.ccbb.utexas.edu/). This alignment was next constrained back to the nucleotide alignment. Some genes (e.g., *infA*, *rpl22*, and *accD*) that got lost from the majority of species were excluded from the analysis. The second data set included the *ycf1* gene sequence from the same taxa as the 78-gene data set except that those taxa without *ycf1* were deleted. These sequences were aligned using the program MAFFT version 5 [25] and adjusted manually where necessary.

Maximum likelihood (ML) analyses were implemented in RAxML version 7.2.6 [53]. RAxML searches relied on the general time reversible (GTR) model of nucleotide substitution with the gamma model of rate heterogeneity. Non-parametric bootstrapping as implemented in the ‘‘fast bootstrap’’ algorithm of RAxML used 1,000 replicates. The aligned data matrices are available upon request. MP analyses were performed with PAUP*4.0b10. Heuristic tree searches were conducted with 1,000 random-taxon-addition replicates and tree bisection-reconnection (TBR) branch swapping, with ‘‘multrees’’ option in effect. Non-parametric bootstrap analysis was conducted under 1,000 replicates with TBR branch swapping.

## Supporting Information

Figure S1 **Visualization of alignments among the five Rosaceae chloroplast genome sequences.** VISTA-based identity plots show sequence identity among the five sequenced chloroplast genomes with *P. persica* as a reference. Genome regions are color-coded as coding and non-coding regions.
(TIF)

Figure S2 **MP phylogram of the angiosperms using whole chloroplast genome sequences.** Numbers above each node indicate the MP bootstrap support values.
(TIF)

Figure S3 **Gene divergences among the five Rosaceae species.** The genes are oriented according to their locations in the chloroplast genome.
(TIF)

Figure S4 **ML phylogram of the angiosperms using the *ycf1* gene sequences.** Numbers at the nodes are ML bootstrap support values.
(TIF)

Figure S5 **ML phylogram of the five Rosaceae species using whole chloroplast genome sequences and ten preferential genes, respectively.** Each tree was conducted using *Morus* *indica* as outgroup. Numbers above each node indicate the ML bootstrap support values.
(TIF)

Table S1 Primers used for validating genome assembly and gaps closing.
(XLS)

Table S2 **Taxa included in the phylogenetic analyses with GenBank accession numbers.**
(DOC)

## References

[1] Potter D, Eriksson T, Evans RC, Oh S, Smedmark J et al. (2007) Phylogeny and classification of Rosaceae. Plant Syst Evol 266: 5-43. doi:10.1007/s00606-007-0539-9.

[2] Potter D, Gao F, Bortiri PE, Oh SH, Baggett S (2002) Phylogenetic relationships in Rosaceae inferred from chloroplast *matK* and *trnL*-*trnF* nucleotide sequence data. Plant Syst Evol 231: 77-89. doi:10.1007/s006060200012.

[3] Kalkman C (2008) The phylogeny of the Rosaceae. Bot J Linn Soc 98: 37-59.

[4] Shulaev V, Sargent DJ, Crowhurst RN, Mockler TC, Folkerts O et al. (2010) The genome of woodland strawberry (*Fragaria vesca*). Nat Genet 43: 109-116. PubMed: 21186353. 21186353 10.1038/ng.740 PMC3326587

[5] Velasco R, Zharkikh A, Affourtit J, Dhingra A, Cestaro A et al. (2010) The genome of the domesticated apple (*Malus* × *domestica* Borkh.). Nat Genet 42: 833-839. doi:10.1038/ng.654. PubMed: 20802477. 20802477 10.1038/ng.654

[6] Wu J, Wang Z, Shi Z, Zhang S, Ming R et al. (2012) The genome of pear (*Pyrus bretschneideri* Rehd.). Genome Res 23: 396-408. PubMed: 23149293. 23149293 10.1101/gr.144311.112 PMC3561880

[7] Zhang Q, Chen W, Sun L, Zhao F, Huang B et al. (2012) The genome of *Prunus mume* . Nat Commun 3: 1318. doi:10.1038/ncomms2290. PubMed: 23271652. 23271652 10.1038/ncomms2290 PMC3535359

[8] Terakami S, Matsumura Y, Kurita K, Kanamori H, Katayose Y et al. (2012) Complete sequence of the chloroplast genome from pear (*Pyrus pyrifoliae*): genome structure and comparative analysis. Tree Genet Genomes: 8: 841-854. doi:10.1007/s11295-012-0469-8.

[9] Jansen RK, Saski C, Lee SB, Hansen AK, Daniell H (2011) Complete plastid genome sequences of three rosids (*Castanea*, *Prunus*, *Theobroma*): evidence for at least two independent transfers of *rpl22* to the nucleus. Mol Biol Evol 28: 835-847. doi:10.1093/molbev/msq261. PubMed: 20935065. 20935065 10.1093/molbev/msq261 PMC3108605

[10] Wu ZY (1986) Flora reipublicae popularis Sinicae. 38. Beijing: Science Press pp. 4-5.

[11] Wu ZY (1995) Flora of Yunnanica. 12. Beijing: Science Press p. 608.

[12] Xu YQ, Yao Z, Hu JY, Teng J, Yoshihisa T et al. (2007) Immunosuppressive terpenes from *Prinsepia utilis* . J Asian Nat Prod Res 9: 637-642. doi:10.1080/10286020600979589. PubMed: 17943558. 17943558 10.1080/10286020600979589

[13] SQ Z, DS Y, XT L, JL L, ZQ Y (2010) Identification and determination of total flavonoid: from Prinsepia utilis Royle. Med Plant 1: 12-15.

[14] Sterling C (1963) The affinities of *Prinsepia* (Rosaceae). Am J Bot: 693-699.

[15] Royle JF, Hope FW, Westwood JO, Ogilby W (1839) Illustrations of the botany and other branches of the natural history of the Himalayan Mountains, and of the flora of Cashmere. Vol. I. London: W. H. Allen and Co..

[16] Lindley J (1836) A Natural System of Botany. 2nd ed. Rees: Longman, Orme, Brown, Green, and London: Longman.

[17] Bentham G, Hooker J (1862–1867) Genera Plantarum. Reeve Co Williams Northgate London. Vol. 1.

[18] Dyall SD, Brown MT, Johnson PJ (2004) Ancient invasions: from endosymbionts to organelles. Science 304: 253–257. doi:10.1126/science.1094884. PubMed: 15073369. 15073369 10.1126/science.1094884

[19] Jansen RK, Raubeson LA, Boore JL, dePamphilis CW, Chumley TW et al. (2005) Methods for obtaining and analyzing whole chloroplast genome sequences. Methods Enzymol 395: 348–384. doi:10.1016/S0076-6879(05)95020-9. PubMed: 15865976. 15865976 10.1016/S0076-6879(05)95020-9

[20] Jansen RK, Cai Z, Raubeson LA, Daniell H, Depamphilis CW et al. (2007) Analysis of 81 genes from 64 plastid genomes resolves relationships in angiosperms and identifies genome-scale evolutionary patterns. Proc Natl Acad Sci U S A 104: 19369-19374. doi:10.1073/pnas.0709121104. PubMed: 18048330. 18048330 10.1073/pnas.0709121104 PMC2148296

[21] Parks M, Cronn R, Liston A (2009) Increasing phylogenetic resolution at low taxonomic levels using massively parallel sequencing of chloroplast genomes. BMC Biol 7: 84. doi:10.1186/1741-7007-7-84. PubMed: 19954512. 19954512 10.1186/1741-7007-7-84 PMC2793254

[22] Moore MJ, Soltis PS, Bell CD, Burleigh JG, Soltis DE (2010) Phylogenetic analysis of 83 plastid genes further resolves the early diversification of eudicots. Proc Natl Acad Sci U S A 107: 4623–4628. doi:10.1073/pnas.0907801107. PubMed: 20176954. 20176954 10.1073/pnas.0907801107 PMC2842043

[23] Shendure J, Ji H (2008) Next-generation DNA sequencing. Nat Biotechnol 26: 1135–1145. doi:10.1038/nbt1486. PubMed: 18846087. 18846087 10.1038/nbt1486

[24] Plunkett GM, Downie SR (2000) Expansion and contraction of the chloroplast inverted repeat in Apiaceae subfamily Apioideae. Syst Bot 25: 648-667. doi:10.2307/2666726.

[25] Katoh K, Kuma K, Toh H, Miyata T (2005) MAFFT version 5: improvement in accuracy of multiple sequence alignment. Nucleic Acids Res 33: 511-518. doi:10.1093/nar/gki198. PubMed: 15661851. 15661851 10.1093/nar/gki198 PMC548345

[26] Clegg MT, Gaut BS, Learn GH, Morton BR (1994) Rates and patterns of chloroplast DNA evolution. Proc Natl Acad Sci U S A 91: 6795-6801. doi:10.1073/pnas.91.15.6795. PubMed: 8041699. 8041699 10.1073/pnas.91.15.6795 PMC44285

[27] Weng ML, Ruhlman TA, Gibby M, Jansen RK (2012) Phylogeny, rate variation, and genome size evolution of *Pelargonium* (Geraniaceae). Mol Phylogenet Evol 64: 654–670. doi:10.1016/j.ympev.2012.05.026. PubMed: 22677167. 22677167 10.1016/j.ympev.2012.05.026

[28] Yi DK, Kim KJ (2012) Complete chloroplast genome sequences of important oilseed crop sesamum indicum L. PLOS ONE 7: e35872. doi:10.1371/journal.pone.0035872. PubMed: 22606240. 22606240 10.1371/journal.pone.0035872 PMC3351433

[29] Powell W, Morgante M, Andre C, McNicol JW, Machray GC et al. (1995) Hypervariable microsatellites provide a general source of polymorphic DNA markers for the chloroplast genome. Curr Biol 5: 1023-1029. doi:10.1016/S0960-9822(95)00206-5. PubMed: 8542278. 8542278 10.1016/s0960-9822(95)00206-5

[30] Bai WN, Liao WJ, Zhang DY (2010) Nuclear and chloroplast DNA phylogeography reveal two refuge areas with asymmetrical gene flow in a temperate walnut tree from East Asia. New Phytol 188: 892-901. doi:10.1111/j.1469-8137.2010.03407.x. PubMed: 20723077. 20723077 10.1111/j.1469-8137.2010.03407.x

[31] Chatrou LW, Escribano MP, Viruel MA, Maas JW, Richardson JE et al. (2009) Flanking regions of monomorphic microsatellite loci provide a new source of data for plant species-level phylogenetics. Mol Phylogenet Evol 53: 726-733. doi:10.1016/j.ympev.2009.07.024. PubMed: 19646541. 19646541 10.1016/j.ympev.2009.07.024

[32] Doorduin L, Gravendeel B, Lammers Y, Ariyurek Y, Chin-A-Woeng T, et al (2011) The complete chloroplast genome of 17 individuals of pest species *Jacobaea vulgaris*: SNPs, microsatellites and bar coding markers for population and phylogenetic studies. DNA Res 18: 93-105. doi:10.1093/dnares/dsr002. PubMed: 21444340. 21444340 10.1093/dnares/dsr002 PMC3077038

[33] Raubeson LA, Peery R, Chumley TW, Dziubek C, Fourcade HM et al. (2007) Comparative chloroplast genomics: analyses including new sequences from the angiosperms *Nuphar advena* and *Ranunculus macranthus* . BMC Genomics 8: 174. doi:10.1186/1471-2164-8-174. PubMed: 17573971. 17573971 10.1186/1471-2164-8-174 PMC1925096

[34] Ellegren H (2004) Microsatellites: simple sequences with complex evolution. Nat Rev Genet 5: 435-445. doi:10.1038/ni1044. PubMed: 15153996. 15153996 10.1038/nrg1348

[35] Rose O, Falush D (1998) A threshold size for microsatellite expansion. Mol Biol Evol 15: 613-615. doi:10.1093/oxfordjournals.molbev.a025964. PubMed: 9580993. 9580993 10.1093/oxfordjournals.molbev.a025964

[36] Levinson G, Gutman GA (1987) Slipped-strand mispairing: a major mechanism for DNA sequence evolution. Mol Biol Evol 4: 203-221. PubMed: 3328815. 3328815 10.1093/oxfordjournals.molbev.a040442

[37] Strand M, Prolla TA, Liskay RM, Petes TD (1993) Destabilization of tracts of simple repetitive DNA in yeast by mutations affecting DNA mismatch repair. Nature 365: 274-276. doi:10.1038/365274a0. PubMed: 8371783. 8371783 10.1038/365274a0

[38] Kuang DY, Wu H, Wang YL, Gao LM, Zhang SZ et al. (2011) Complete chloroplast genome sequence of Magnolia *kwangsiensis* (Magnoliaceae): implication for DNA bar coding and population genetics. Genome 54: 663-673. doi:10.1139/g11-026. PubMed: 21793699. 21793699 10.1139/g11-026

[39] Cai Z, Guisinger M, Kim HG, Ruck E, Blazier JC et al. (2008) Extensive reorganization of the plastid genome of *Trifolium subterraneum* (Fabaceae) is associated with numerous repeated sequences and novel DNA insertions. J Mol Evol 67: 696-704. doi:10.1007/s00239-008-9180-7. PubMed: 19018585. 19018585 10.1007/s00239-008-9180-7

[40] Moore MJ, Bell CD, Soltis PS, Soltis DE (2007) Using plastid genome-scale data to resolve enigmatic relationships among basal angiosperms. Proc Natl Acad Sci U S A 104: 19363-19368. doi:10.1073/pnas.0708072104. PubMed: 18048334. 18048334 10.1073/pnas.0708072104 PMC2148295

[41] Bapteste E, Philippe H (2002) The potential value of indels as phylogenetic markers: position of trichomonads as a case study. Mol Biol Evol 19: 972-977. doi:10.1093/oxfordjournals.molbev.a004156. PubMed: 12032255. 12032255 10.1093/oxfordjournals.molbev.a004156

[42] Egan AN, Crandall KA (2008) Incorporating gaps as phylogenetic characters across eight DNA regions: Ramifications for North American Psoraleeae (Leguminosae). Mol Phylogenet Evol 46: 532-546. doi:10.1016/j.ympev.2007.10.006. PubMed: 18039582. 18039582 10.1016/j.ympev.2007.10.006

[43] Neubig KM, Whitten WM, Carlsward BS, Blanco MA, Endara L et al. (2009) Phylogenetic utility of *ycf1* in orchids: a plastid gene more variable than *matK* . Plant Syst Evol 277: 75-84. doi:10.1007/s00606-008-0105-0.

[44] Gernandt DS, Hernández-León S, Salgado-Hernández E, Rosa JAP (2009) Phylogenetic relationships of *Pinus* subsection *Ponderosae* inferred from rapidly evolving cpDNA regions. Syst Bot 34: 481-491. doi:10.1600/036364409789271290.

[45] Neubig KM, Abbott JR (2010) Primer development for the plastid region *ycf1* in Annonaceae and other magnoliids. Am J Bot 97: e52-e55. doi:10.3732/ajb.1000128. PubMed: 21622459. 21622459 10.3732/ajb.1000128

[46] Shi C, Hu N, Huang H, Gao J, Zhao YJ et al. (2012) An improved chloroplast DNA extraction procedure for whole plastid genome sequencing. PLOS ONE 7: e31468. doi:10.1371/journal.pone.0031468. PubMed: 22384027. 22384027 10.1371/journal.pone.0031468 PMC3285163

[47] Li R, Zhu H, Ruan J, Qian W, Fang X et al. (2010) De novo assembly of human genomes with massively parallel short read sequencing. Genome Res 20: 265-272. doi:10.1101/gr.097261.109. PubMed: 20019144. 20019144 10.1101/gr.097261.109 PMC2813482

[48] Shaw J, Lickey EB, Schilling EE, Small RL (2007) Comparison of whole chloroplast genome sequences to choose noncoding regions for phylogenetic studies in angiosperms: the tortoise and the hare III. Am J Bot 94: 275-288. doi:10.3732/ajb.94.3.275. PubMed: 21636401. 21636401 10.3732/ajb.94.3.275

[49] Straub SCK, Parks M, Weitemier K, Fishbein M, Cronn RC et al. (2012) Navigating the tip of the genomic iceberg: Next-generation sequencing for plant systematics. Am J Bot 99: 349-364. doi:10.3732/ajb.1100335. PubMed: 22174336. 22174336 10.3732/ajb.1100335

[50] Wyman SK, Jansen RK, Boore JL (2004) Automatic annotation of organellar genomes with DOGMA. Bioinformatics 20: 3252-3255. doi:10.1093/bioinformatics/bth352. PubMed: 15180927. 15180927 10.1093/bioinformatics/bth352

[51] Lohse M, Drechsel O, Bock R (2007) Organellar Genome DRAW (OGDRAW): a tool for the easy generation of high-quality custom graphical maps of plastid and mitochondrial genomes. Curr Genet 52: 267-274. doi:10.1007/s00294-007-0161-y. PubMed: 17957369. 17957369 10.1007/s00294-007-0161-y

[52] Tamura K, Peterson D, Peterson N, Stecher G, Nei M et al. (2011) MEGA5: molecular evolutionary genetics analysis using maximum likelihood, evolutionary distance, and maximum parsimony methods. Mol Biol Evol 28: 2731-2739. doi:10.1093/molbev/msr121. PubMed: 21546353. 21546353 10.1093/molbev/msr121 PMC3203626

[53] Stamatakis A (2006) RAxML-VI-HPC: maximum likelihood-based phylogenetic analyses with thousands of taxa and mixed models. Bioinformatics 22: 2688-2690. doi:10.1093/bioinformatics/btl446. PubMed: 16928733. 16928733 10.1093/bioinformatics/btl446

