# Supplementary figures and images for: Plastid Genome Sequence of a Wild Woody Oil Species, Prinsepia utilis, Provides Insights into Evolutionary and Mutational Patterns of Rosaceae Chloroplast Genomes
Source: PLoS One. 2013 Sep 2;8(9):e73946. doi: 10.1371/journal.pone.0073946 (PMC3759469; doi:10.1371/journal.pone.0073946)

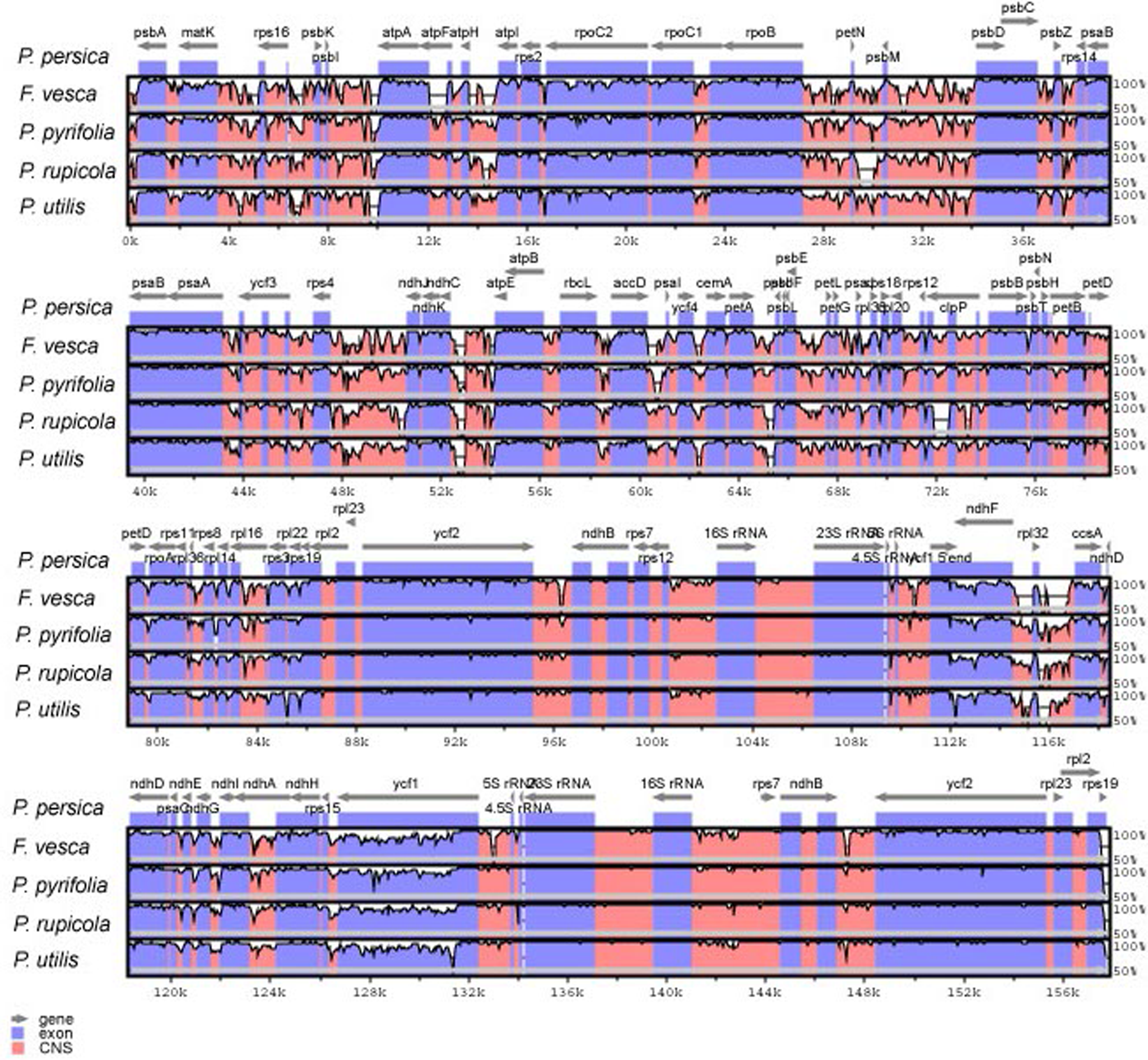

Supplement: Figure S1 — Visualization of alignments among the five Rosaceae chloroplast genome sequences. VISTA-based identity plots show sequence identity among the five sequenced chloroplast genomes with P. persica as a reference. Genome regions are color-coded as coding and non-coding regions. (TIF) [file pone.0073946.s001.tif]

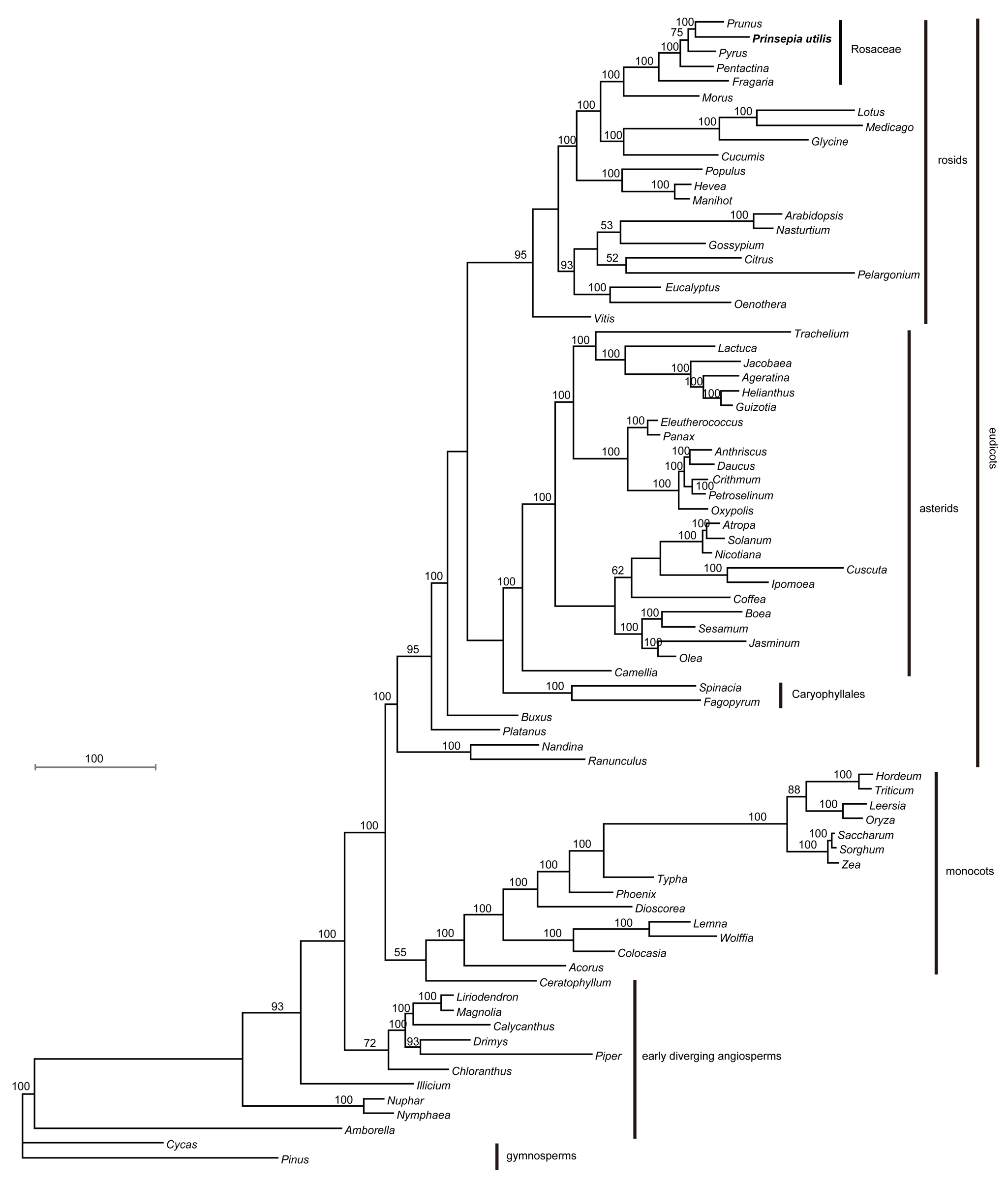

Supplement: Figure S2 — MP phylogram of the angiosperms using whole chloroplast genome sequences. Numbers above each node indicate the MP bootstrap support values. (TIF) [file pone.0073946.s002.tif]

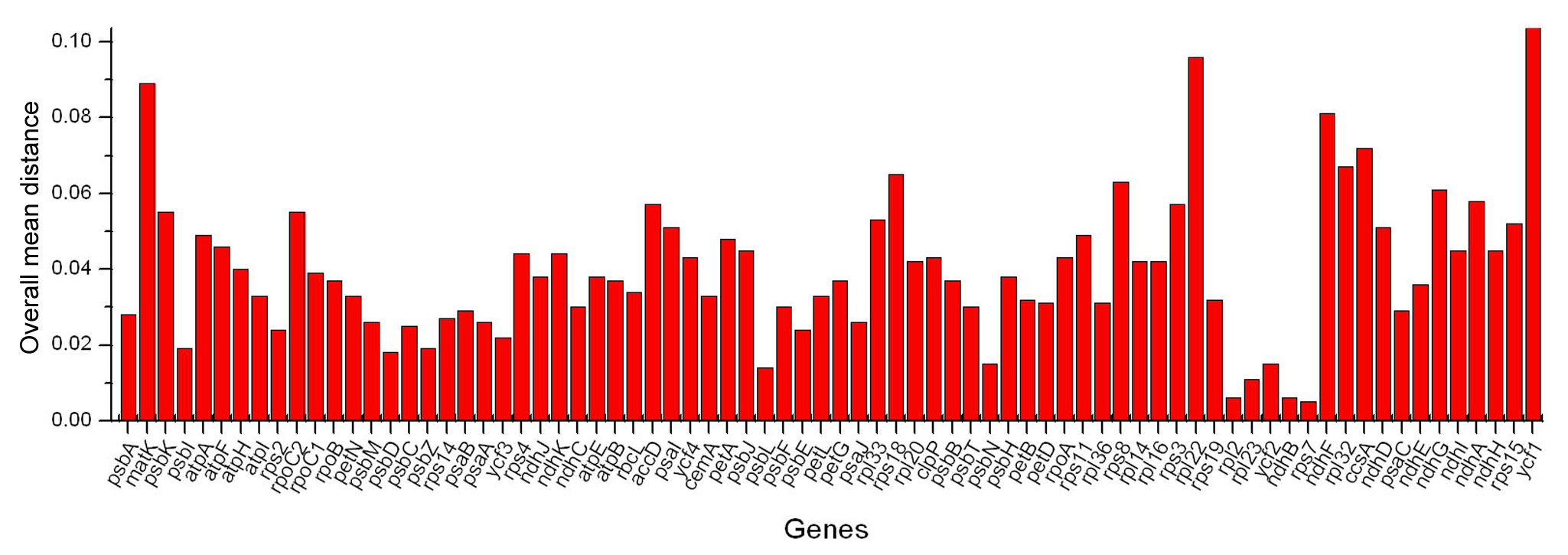

Supplement: Figure S3 — Gene divergences among the five Rosaceae species. The genes are oriented according to their locations in the chloroplast genome. (TIF) [file pone.0073946.s003.tif]

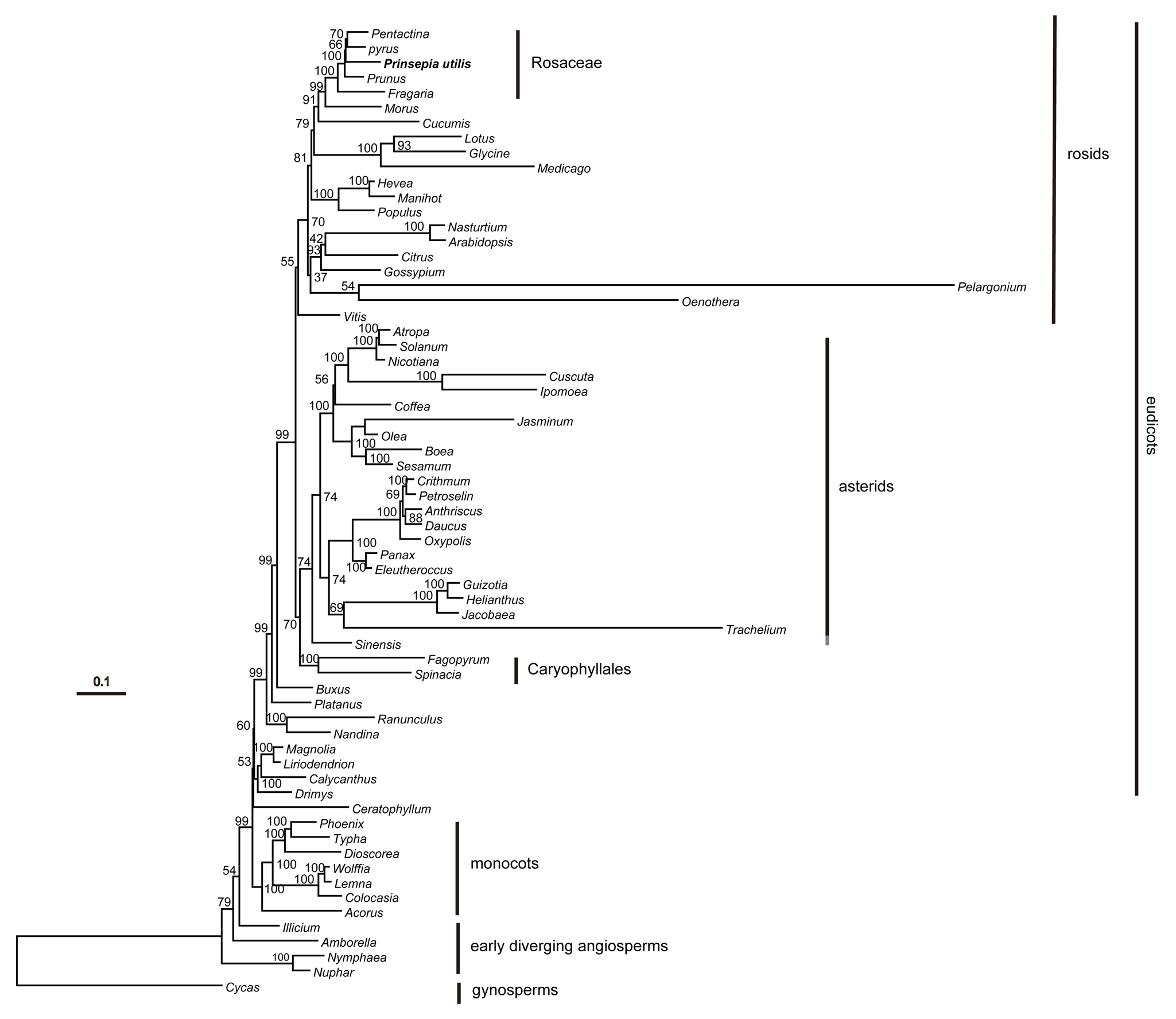

Supplement: Figure S4 — ML phylogram of the angiosperms using the ycf1 gene sequences. Numbers at the nodes are ML bootstrap support values. (TIF) [file pone.0073946.s004.tif]

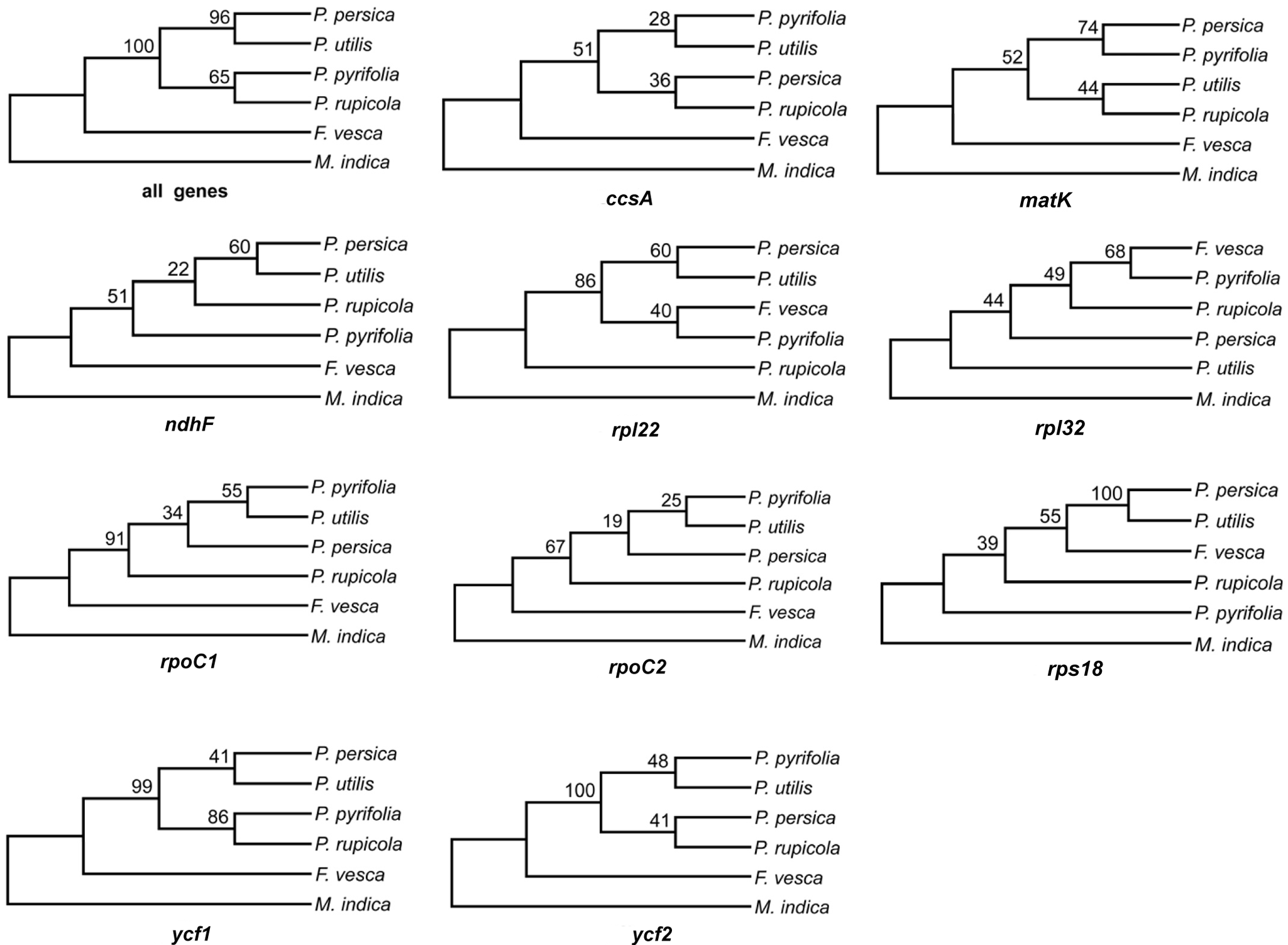

Supplement: Figure S5 — ML phylogram of the five Rosaceae species using whole chloroplast genome sequences and ten preferential genes, respectively. Each tree was conducted using Morus indica as outgroup. Numbers above each node indicate the ML bootstrap support values. (TIF) [file pone.0073946.s005.tif]
