# Supplementary material for: Plastid Genome Sequence of a Wild Woody Oil Species, Prinsepia utilis, Provides Insights into Evolutionary and Mutational Patterns of Rosaceae Chloroplast Genomes
Source: PLoS One. 2013 Sep 2;8(9):e73946. doi: 10.1371/journal.pone.0073946 (PMC3759469; doi:10.1371/journal.pone.0073946)
Supplement: Table S2 — Taxa included in the phylogenetic analyses with GenBank accession numbers. (DOC) [file pone.0073946.s007.doc]

**Additional Table 2 – Taxa included in the phylogenetic analyses with GenBank accession numbers.**

| **Classification** | **Taxon** | **Genbank accessions** |
| --- | --- | --- |
| **Gymnosperms** |  |  |
|  | *Pinus thunbergii* | NC_001631 |
|  | *Cycas taitungensis* | NC_009618 |
| **Early diverging groups** |  |  |
|  | *Amborella trichopoda* | NC_005086 |
|  | *Nymphaea alba* | NC_006050 |
|  | *Nuphar advena* | NC_008788 |
|  | *Illicium oligandrum* | NC_009600 |
|  | *Chloranthus spicatus* | NC_009598 |
|  | *Drimys granadensis* | NC_008456 |
|  | *Piper cenocladum* | NC_008457 |
|  | *Calycanthus floridus* var. *glaucus* | NC_004993 |
|  | *Magnolia kwangsiensis* | NC_015892 |
|  | *Liriodendron tulipifera* | NC_008326 |
|  | *Nandina domestica* | NC_008336 |
|  | *Ranunculus macranthus* | NC_008796 |
|  | *Platanus occidentalis* | NC_008335 |
|  | *Buxus microphylla* | NC_009599 |
|  | *Ceratophyllum demersum* | NC_009962 |
| **Monocots** |  |  |
|  | *Acorus calamus* | NC_007407 |
|  | *Wolffia australiana* | NC_015899 |
|  | *Lemna minor* | NC_010109 |
|  | *Phalaenopsis aphrodite* | NC_007499 |
|  | *Dioscorea elephantipes* | NC_009601 |
|  | *Phoenix dactylifera* | NC_013991 |
|  | *Typha latifolia* | NC_013823 |
|  | *Oryza nivara* | NC_005973 |
|  | *Triticum aestivum* | NC_002762 |
|  | *Hordeum vulgare* | NC_008590 |
|  | *Zea mays* | NC_001666 |
|  | *Sorghum bicolor* | NC_008602 |
|  | *Saccharum* hybrid cultivar SP-80-3280 | NC_005878 |
|  | *Leersia tisserantii* | NC_016677 |
| **Rosids** |  |  |
|  | *Vitis vinifera* | NC_007957 |
|  | *Populus alba* | NC_008235 |
|  | *Hevea brasiliensis* | NC_015308 |
|  | *Manihot esculenta* | NC_010433 |
|  | *Cucumis sativus* | NC_007144 |
|  | *Prunus persica* | NC_014697 |
|  | *Morus indica* | NC_008359 |
|  | *Glycine max* | NC_007942 |
|  | *Lotus japonicus* | NC_002694 |
|  | *Medicago truncatula* | NC_003119 |
|  | *Eucalyptus grandis* | NC_014570 |
|  | *Oenothera argillicola* | NC_010358 |
|  | *Pelargonium x hortorum* | NC_008454 |
|  | *Citrus sinensis* | NC_008334 |
|  | *Gossypium arboreum* | NC_016712 |
|  | *Nasturtium officinale* | NC_009275 |
|  | *Arabidopsis thaliana* | NC_000932 |
|  | *Pyrus pyrifolia* | NC_015996 |
|  | *Pentactina rupicola* | NC_016921 |
|  | *Fragaria vesca* | NC_015206 |
|  | ***Prinsepia utilis*** | **KC571835** |
| **Caryophyllales** |  |  |
|  | *Spinacia oleracea* | NC_002202 |
|  | *Fagopyrum esculentum* | NC_010776 |
| **Asterids** |  |  |
|  | *Camellia sinensis* var. *assamica* | JQ975030 |
|  | *Coffea arabica* | NC_008535 |
|  | *Jasminum nudiflorum* | NC_008407 |
|  | *Olea europaea* | NC_013707 |
|  | *Boea hygrometrica* | NC_016468 |
|  | *Sesamum indicum* | NC_016433 |
|  | *Ipomoea purpurea* | NC_009808 |
|  | *Cuscuta exaltata* | NC_009963 |
|  | *Nicotiana tabacum* | NC_001879 |
|  | *Atropa belladonna* | NC_004561 |
|  | *Solanum bulbocastanum* | NC_007943 |
|  | *Panax ginseng* | NC_006290 |
|  | *Eleutherococcus senticosus* | NC_016430 |
|  | *Oxypolis greenmanii* | NC_015832 |
|  | *Crithmum maritimum* | NC_015804 |
|  | *Petroselinum crispum* | NC_015821 |
|  | *Anthriscus cerefolium* | NC_015113 |
|  | *Daucus carota* | NC_008325 |
|  | *Trachelium caeruleum* | NC_010442 |
|  | *Lactuca sativa* | NC_007578 |
|  | *Jacobaea vulgaris* | NC_015543 |
|  | *Ageratina adenophora* | NC_015621 |
|  | *Helianthus annuus* | NC_007977 |
|  | *Guizotia abyssinica* | NC_010601 |
